# Development of severe intrapulmonary shunting in a patient with carcinoid heart disease after closure of a persistent foramen ovale: a case report

**DOI:** 10.1093/ehjcr/ytab494

**Published:** 2021-12-04

**Authors:** Dominik Schüttler, Konstantinos Mourouzis, Christoph J Auernhammer, Konstantinos D Rizas

**Affiliations:** 1 Department of Medicine I, University Hospital, LMU Munich, Marchioninistr. 15, 81377 Munich, Germany; 2 DZHK (German Centre for Cardiovascular Research), Partner Site Munich, Munich Heart Alliance (MHA), Munich, Germany; 3 Walter Brendel Centre of Experimental Medicine, Ludwig-Maximilians-University Munich (LMU), Marchioninistr. 15, 81377 Munich, Germany; 4 Department of Internal Medicine IV, University Hospital Munich (LMU) Campus Grosshadern, Marchioninistr. 15, 81377 Munich, Germany; 5 Interdisciplinary Center of Neuroendocrine Tumours of the GastroEnteroPancreatic System (GEPNET-KUM, ENETS Center of Excellence), University Hospital Munich (LMU), Marchioninistr. 15, 81377 Munich, Germany

**Keywords:** Neuroendocrine tumour, Carcinoid heart disease, Hedinger syndrome, Carcinoid valve disease, Patent foramen ovale, Pulmonary shunt, Case report

## Abstract

**Background:**

Neuroendocrine tumours (NETs) can affect the cardiopulmonary system causing carcinoid heart disease (CHD) and valve destruction. Persistent foramen ovale (PFO) occlusion is indicated in patients with CHD and shunt-related left heart valve involvement.

**Case summary:**

We report the case of a 54-year-old female patient with metastatic NET originating from the small bowel. The patient was on medication with octreotide and telotristat. One year after diagnosis, cardiac involvement of carcinoid developed with regurgitation of right-sided and, due to PFO, left-sided heart valves. Closure of PFO was performed (Occlutech 16/18 mm). One year later, she presented with recurrent severe dyspnoea. The PFO occluder was *in situ* without residual shunt. Valvular heart disease, including left-sided disease, and metastatic spread of NET were stable. Blood gas analysis revealed arterial hypoxaemia (pO_2_ = 44 mmHg/5.87 kPa), which was related to extensive intrapulmonary shunting (31% shunt fraction) confirmed using contrast-enhanced echocardiography. The patient was prescribed long-term oxygen supplementation as symptomatic therapy and anti-tumoural therapy was intensified with selective internal radiotherapy (SIRT) of the liver metastases to improve biochemical control of the carcinoid syndrome. At a follow-up visit 4 months after SIRT, the patient-reported stable dyspnoea; however, magnetic resonance imaging revealed progression of osseous metastases.

**Discussion:**

An echocardiographic assessment of the presence of a PFO is recommended in patients with NET as PFO closure minimizes the risk of left-sided carcinoid valve disease. Deterioration of symptomatic status in metastasized NET might also be due to a hepatopulmonary-like physiology with intrapulmonary shunting and arterial desaturation thought to be caused by vasoactive substances secreted by the tumour. This is a rare case describing the development of this syndrome after PFO closure.

Learning points

- Functionally active neuroendocrine tumours (NETs) with carcinoid syndrome can cause carcinoid heart disease with cardiac valve destruction and dysfunction.
- Involvement of left-sided valves is often related to patent foramen ovale (PFO) allowing shunting of vasoactive substances due to elevated right atrial pressure which can be minimized by transcatheter closure of PFO.
- NETs are bioactive tumours secreting vasoactive substances which on rare occasions can disrupt vascular tone in the pulmonary circulation causing a hepatopulmonary-like physiology without necessarily liver dysfunction.
- This is one of the few case reports describing the development of severe intrapulmonary shunting in carcinoid disease, and one of the very few cases of hepatopulmonary-like syndrome without significant liver dysfunction.

## Introduction

Neuroendocrine tumours (NETs) are rare neoplastic disorders. The overall prognosis depends on the originating site, as well as the stage and grade of the disease at the time of diagnosis.^1^ Neuroendocrine tumours may secrete vasoactive substances, biogenic amines, and peptide hormones, most importantly serotonin.^2^ This is considered one of the main pathophysiological steps for the development of carcinoid heart disease (CHD, Hedinger syndrome) which occurs, with or without metastatic liver involvement,^3^ in 20–40% of all functionally active NETs with carcinoid syndrome (CS).^4^ Carcinoid heart disease leads to fibrotic thickening of valves with subsequent destruction, stenosis, and/or regurgitation of right-sided valves.^2^ The presence of a patent foramen ovale (PFO) can lead to an expansion of the disease to left-sided valves, as atrial right-to-left shunting may be relevant due to high right atrial pressures subsequent to right-sided valve disease. Transcatheter closure of PFO has been demonstrated to improve symptoms and progression of left-sided valve destruction.^5^ Severe liver dysfunction can cause hepatopulmonary syndrome (HPS) with intrapulmonary vasodilation, which leads to abnormal low blood oxygenation due to shunting.^6^ However, this is mostly seen at a cirrhotic stage.^7^ Here, we report the case of a patient with NET, who developed a hepatopulmonary-like physiology without noticeable liver dysfunction in the context of CHD and PFO.

## Timeline

**Table:** 

Baseline month 0: Diagnosis of metastatic neuroendocrine tumour (liver biopsy, G2, Ki-67 3%), initiation of therapy with octreotide. Follow-up months 1–8: Four cycles of ^177^Lutetium-DOTA-TATE therapy, a peptide receptor radionuclide therapy targeting somatostatin receptors. Follow-up month 8: Diagnosis of carcinoid heart disease with severe tricuspid valve insufficiency; detection of persistent foramen ovale (PFO). Follow-up month 12: Development of both mitral and aortic valve regurgitation. Transcatheter closure of PFO (Occlutech 16/18 mm). Follow-up month 21: Initiation of therapy with telotristat, an inhibitor of tryptophan hydroxylase in response to persisting high serotonin levels. Follow-up month 22: Recurrence of severe dyspnoea by detection of severe intrapulmonary shunting. Initiation of long-term oxygen therapy and selective internal radiotherapy of liver metastases. Follow-up month 28: Stable dyspnoea but progression of osseous metastases.

## Case presentation

A 54-year-old Caucasian female patient without prior medical history was initially admitted to our hospital with severe fatigue, intermittent diarrhoea, and flushing. The patient showed no signs of heart failure, especially no peripheral oedema or jugular vein distention. Heart auscultation was normal without murmurs. Eventually, a NET originating from the small bowel was diagnosed with metastases in liver, lungs, bones, as well as cardio-phrenic and para-aortic lymph nodes. The tumour marker chromogranin A was elevated with 1700 ng/mL (18 × upper limit of normal (ULN)). The NET was functionally active with CS [serotonin in serum 3072 ng/mL (16 × ULN), serotonin metabolite 5-hydroxy-indol-acetic-acid (5-HIAA) in 24 h-urine 192 mg/24 h (24 × ULN)]. Assessment of liver function showed normal transaminases, γ-glutamyltransferase, international normalized ratio (INR), and lactate dehydrogenase and an elevated total bilirubin level (1.9 mg/dL). At this time, the patient was on no cardiac medication. The patient received a biotherapy with the somatostatin receptor analogue octreotide and underwent four cycles of somatostatin receptor-mediated peptide receptor radionuclide therapy with ^177^Lutetium-DOTA-TATE in order to improve biochemical and symptom control of the CS and to achieve tumour control.^4^

One year later, the patient reported increasing dyspnoea on exertion. A peripheral oxygen saturation of 90–92% on room air was measured at that time. The diagnosis of a progressive carcinoid valve disease was established by echocardiography. While initially affecting only the right-sided valves, progressive regurgitation of the aortic and mitral valves suggested involvement of the left-sided valves possibly due to right to left shunt through the patent open foramen ovale. As rising pressure in the right atrium due to deterioration of right sides valvular and ventricular function leads to progressive atrial right-to-left shunt, closure of a PFO should be helpful to minimize shunting and deterioration of left-sided valve function. Consequently, the PFO was occluded by a percutaneous closure device (Occlutech PFO occluder 16/18 mm) following current guidelines. After device implantation, no residual shunt was detectable by transoesophageal echocardiography. There was no additional atrial septal defect. A peripheral oxygen saturation of 90–93% on room air was documented after PFO occlusion. 5-hydroxy-indol-acetic-acid levels were stable during that time. The patient was discharged and reported an improvement of symptoms and quality of life throughout the following months.

**Figure 1:**
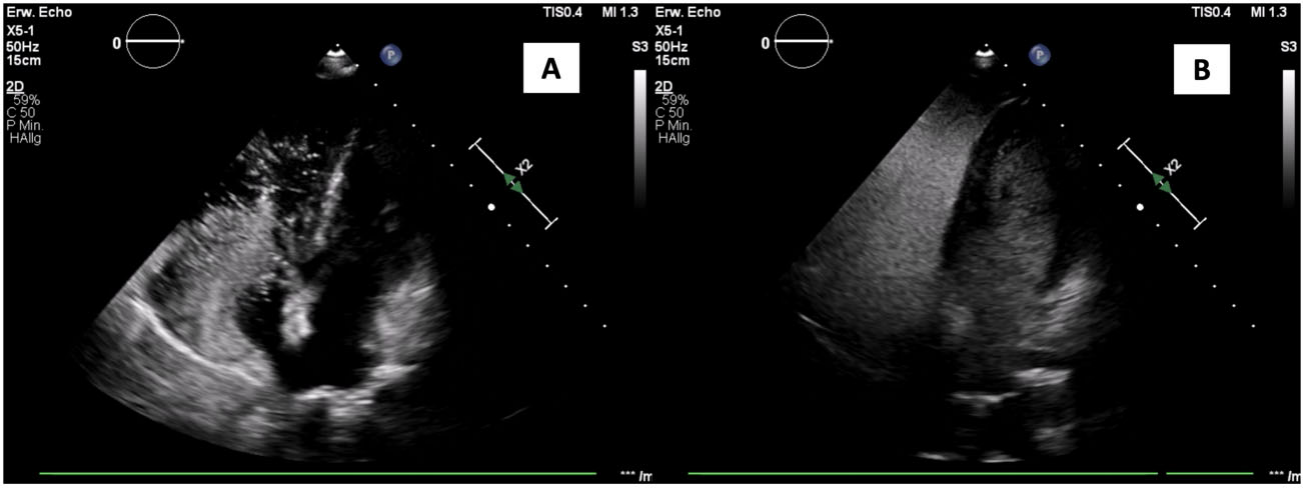
Transthoracic echocardiography with peripheral injection of non-transpulmonary contrast agent. Persistent foramen ovale closure device *in situ* without residual (early) interatrial shunt (*A*). Delayed appearance of microbubbles in the left atrium and ventricle after three to four heart beats (*B*) indicating massive intrapulmonary shunting.

However, her symptoms of dyspnoea began to severely aggravate about 10 months later, which subsequently led to the next hospital admission. At this time, the patient was on cardiac medication with acetyl salicylic acid (Aspirin) 100 mg (after PFO occlusion) and with 5 mg Ramipril due to hypertension once daily. A peripheral oxygen saturation of 82% on room air was measured. Signs of right- and left heart failures such as ascites and pleural effusions were not seen. Initial assessment of liver function demonstrated normal alkaline phosphatase (84 U/L), aspartate transaminase (29 U/L), alanine transaminase (27 U/L), and slightly elevated levels of lactate dehydrogenase (283 U/L). A normal albumin level (3.5 g/dL) and normal INR (1.0) indicated a preserved hepatic functional capacity. Values of γ-glutamyltransferase (118 U/L) and total bilirubin (2.1 mg/dL) were slightly elevated yet decreased compared to previous measurements. A computed tomography (CT) depicted an unchanged distribution and extension of the metastatic loci in lungs, bones, liver, and lymph nodes compared to 3 months ago. Acute pulmonary embolism was excluded by CT pulmonary angiography. A pulmonary ventilation perfusion scintigraphy yielded no evidence for chronic thromboembolic pulmonary hypertension (PH). Right heart catheterization revealed normal pulmonary arterial pressure and pulmonary capillary wedge pressure (*Table 1*) with no step in oxygen saturation between right atrium, right ventricle and pulmonary artery (55% each). Pulmonary function testing showed no signs of obstructive or restrictive lung disease [forced vital capacity (FVC) 3.33 L, forced expiratory volume in 1 s (FEV_1_) 2.65 L, FEV_1_/FVC 81% and diffusing capacity for carbon monoxide 5.83 mmol CO × min^−1^ × kPa^−1^ × m^−2^, corresponding to a 79% of the predicted value]. Arterial blood gas analysis revealed a pH of 7.51, a pCO_2_ of 23 mmHg/3.07 kPa, a HCO3^−^ concentration of 22 mmol/L, and a pO_2_ of 44 mmHg/5.87 kPa. Breathing with 100% supplemental oxygen did not change arterial blood oxygen content (pH 7.47, pCO_2_ 26 mmHg/3.47 kPa, and pO_2_ 50 mmHg/6.67 kPa). By applying the Berggren shunt equation,^8^^,^^9^ a shunt fraction of 31% was calculated. Moreover, transthoracic echocardiography with the injection of a non-transpulmonary contrast agent revealed a delayed and massive microbubble opacification of the left atrium and ventricle, compatible with pronounced intrapulmonary shunting (*Figure 1*, *Video 1*). The PFO closure device was demonstrated *in situ* with no sign of residual leaks, excluding an interatrial cardiac shunt.

**Table 1:** Right heart catheterization with pressure values for pulmonary capillary wedge pressure (PCWP), pulmonary artery (PA), right ventricle (RV), right atrium (RA), pulmonary vascular resistance (PVR), and Wood Units (WU)

	Values
PCWP *a*-*x*-*v*-*y* (mean)	10-9-11-19 (9) mmHg
PA sys/diast (mean)	22/10 (14) mmHg
RV syst/early diast. (mean)	30/7 (11) mmHg
RA *a*-*x*-*v*-*y* (mean)	18-9-20-8 (15) mmHg
PVR	1.8 WU
Cardiac output	2.8 L/min
Cardiac index	2.0 L/min/m^2^

Of note, serotonin and 5-HIAA in 24 h urine were substantially improved compared to baseline but still elevated with 1124 ng/mL (5.8 × ULN) and 42.3 mg/24 h (5.3 × ULN), respectively. Therefore, the tryptophan hydroxylase inhibitor telotristat was added to further improve biochemical and symptom control which subsequently caused a decrease of serotonin and 5-HIAA to 860 ng/mL (4.5 × ULN) and 18.3 mg/24 h (2.3 × ULN). A biochemical progress of metastatic carcinoid disease with increases of tumour-released vasoactive humoral factors towards the pulmonary vasculature were considered as probable cause for the development of this severe intrapulmonary shunting. Therefore, the patient was advised to use long-term oxygen supplementation as symptomatic therapy^7^^,^^10^ and to selective internal radiation therapy of the hepatic metastases with additional octreotide therapy. At a follow-up visit 4 months after selective internal radiotherapy, the patient-reported stable dyspnoea; however, magnetic resonance imaging revealed progression of osseous metastases.

## Discussion

In this case, a patient with known metastatic NET, CHD, and closed PFO, presented with pronounced aggravation of dyspnoeic symptoms combined with a marked reduction of arterial oxygen content, which could not be reverted by supplemental oxygen. Both liver dysfunction as well as macroscopic progression of liver metastases could be excluded. We believe that the relapse of the symptoms can be attributed to a newly developed severe intrapulmonary shunting.

Severe intrapulmonary shunting, considered to be a manifestation of a hepato-pulmonary-like syndrome, has been described in some rare cases of patients with NET.^11^ Originally, HPS consists of the triad of pronounced liver dysfunction, intrapulmonary vascular dilation, and abnormal blood oxygenation.^6^ The pathophysiology of the HPS lies on the impaired catabolism of regulators of the vascular tone, which in turn cause vasodilation of pulmonary veins leading to intrapulmonary shunting.^6^^,^^12^ However, liver function was preserved in our patient while she presented with a hepatopulmonary-like physiology. Most probably this condition resulted from the massive production of vasoactive substances exceeding the catabolic capacity of the liver. This is described to occur at very rare occasions in patients with NETs.^11^ It is suggested that substances secreted from the metastatic carcinoid, which are known to act as vasoactive mediators, result in the observed intrapulmonary shunting and in pronounced arterial hypoxaemia refractory to oxygen supply. Our hypothesis of a hepatopulmonary-like physiology could be confirmed using contrast-enhanced echocardiography, which demonstrated delayed and massive microbubble opacification of the left atrium and ventricle, compatible with a pronounced intrapulmonary shunt in the setting of a CHD.

The pulmonary vasodilation can be attributed to vascular mediators such circulating serotonin and chromogranin. Even though 25% of carcinoid patients suffer from PH,^13^ our patient had no evidence of PH. And although serotonin is primarily linked to vasoconstriction, this case and further evidence shows that serotonin can also lead to vasodilative effects in patients with carcinoid.^14^^,^^15^ Besides serotonin, various bioactive substances are known to evolve from NETs such as vasointestinal peptide, bradykinin, tachykinins, NO, and substance P and these are connected to vasodilatory properties.^16^ Thus, a prolonged exposure to serotonin and these bioactive substances might have led to a cumulative damage of the pulmonary vascular tone and triggered a hepatopulmonary-like physiology in our patient. As serotonin levels decreased while the pulmonary shunting developed, a change in the concentration profile of those vasoactive substances not routinely quantified is likely to largely have contributed.^6^^,^^17^

Progressive dyspnoea in carcinoid disease seen in the setting of a PFO is much more often related to the development of left-sided valve disease which is caused by right-to-left shunting due to a rise in right trial pressure in right-sided heart failure. Here, an interventional closure of PFO is associated with clinical improvement.^5^^,^^18–20^ As our patient also demonstrated right-sided and subsequently, also left-sided valve disease, an occluder device was used to prevent further right-to-left shunting through the PFO. Noteworthy, a massive increase in the release of vasoactive substances also can lead to an involvement of left-sided valves even without the presence of a PFO.^21^ In our case, 5-HIAA levels remained stable at that time. Nevertheless, it cannot be ruled out that the blood levels of other bioactive substances which are not measured in clinical routine may already have risen at the time of PFO closure. Occlusion led to an initial clinical remission. As the shunt fraction via PFO was low in our patient, we do not assume that closure of the PFO significantly accelerated the development of the hepato-pulmonary-like syndrome as seen months later. Nevertheless, we would like to point out that this could well be the case in patients with larger shunt fractions such as with atrial septal defects.

The development of severe pulmonary shunting in patients with CHD is extremely rare. Only few reports are mentioned in the literature describing a similar pathophysiology. Robert *et al*.^22^ and Hussain *et al*.^23^ described cases of carcinoid with hepatopulmonary-like physiology yet in the presence of severe liver dysfunction. Lee and Lepler^11^ described a case similar to ours, suggesting that a long-lasting carcinoid disease could gradually lead to the development of HPS-like manifestations, without necessarily signs of established liver cirrhosis. This is a very rare case of severe intrapulmonary shunting in a patient suffering from CHD, in our patient some months after transcatheter closure of the PFO, and one of the very rare cases of HPS-like syndrome in the absence of significant liver dysfunction.

In summary, based on this report, in patients with CHD, assessment of the presence of a PFO is recommended as PFO closure would be expected to minimize the risk of left-sided carcinoid valve disease. Deterioration of symptomatic status in metastasized NET might also be due to an HPS-like physiology with intrapulmonary shunting and arterial desaturation.

## Lead author biography

**Figure:**
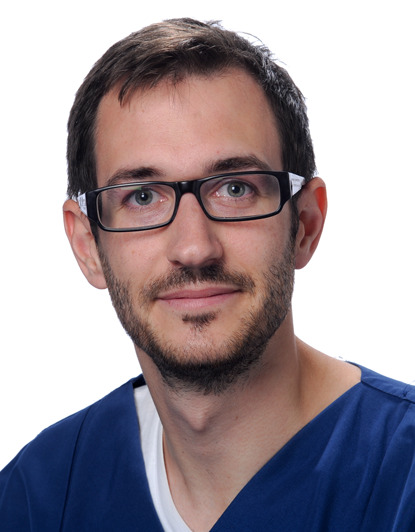


Dominik Schüttler is working at the Department of Cardiology at the University Hospital of Munich (LMU). His research focuses on the pathophysiological mechanisms of atrial fibrillation in large animal models.

## Supplementary material

Supplementary material is available at *European Heart Journal - Case Reports* online.

**Slide sets:** A fully edited slide set detailing this case and suitable for local presentation is available online as Supplementary data.

**Consent:** The authors confirm that written consent for submission and publication of this case report including images and associated text has been obtained from the patient in line with COPE guidance.

**Conflict of interest:** C.J.A. has received research contracts (Ipsen, Novartis), lecture honorarium (AAA, Ipse, Novartis) and advisory board honorarium (Novartis). There is no potential conflict of interest regarding any part of this manuscript.

**Funding:** D.S. is funded by the Deutsche Forschungsgemeinschaft (DFG, German Research Foundation) - 413635475 - and the Munich Clinician Scientist Program (MCSP) of the LMU Munich. The funder had no influence regarding any part of this manuscript.

## Supplementary Material

ytab494_Supplementary_Data
