# Supplementary material for: Development of severe intrapulmonary shunting in a patient with carcinoid heart disease after closure of a persistent foramen ovale: a case report
Source: Eur Heart J Case Rep. 2021 Dec 4;5(12):ytab494. doi: 10.1093/ehjcr/ytab494 (PMC8759518; doi:10.1093/ehjcr/ytab494)
Supplement: ytab494_Supplementary_Data [file ytab494_Supplementary_Data.pptx]

## Slide 1
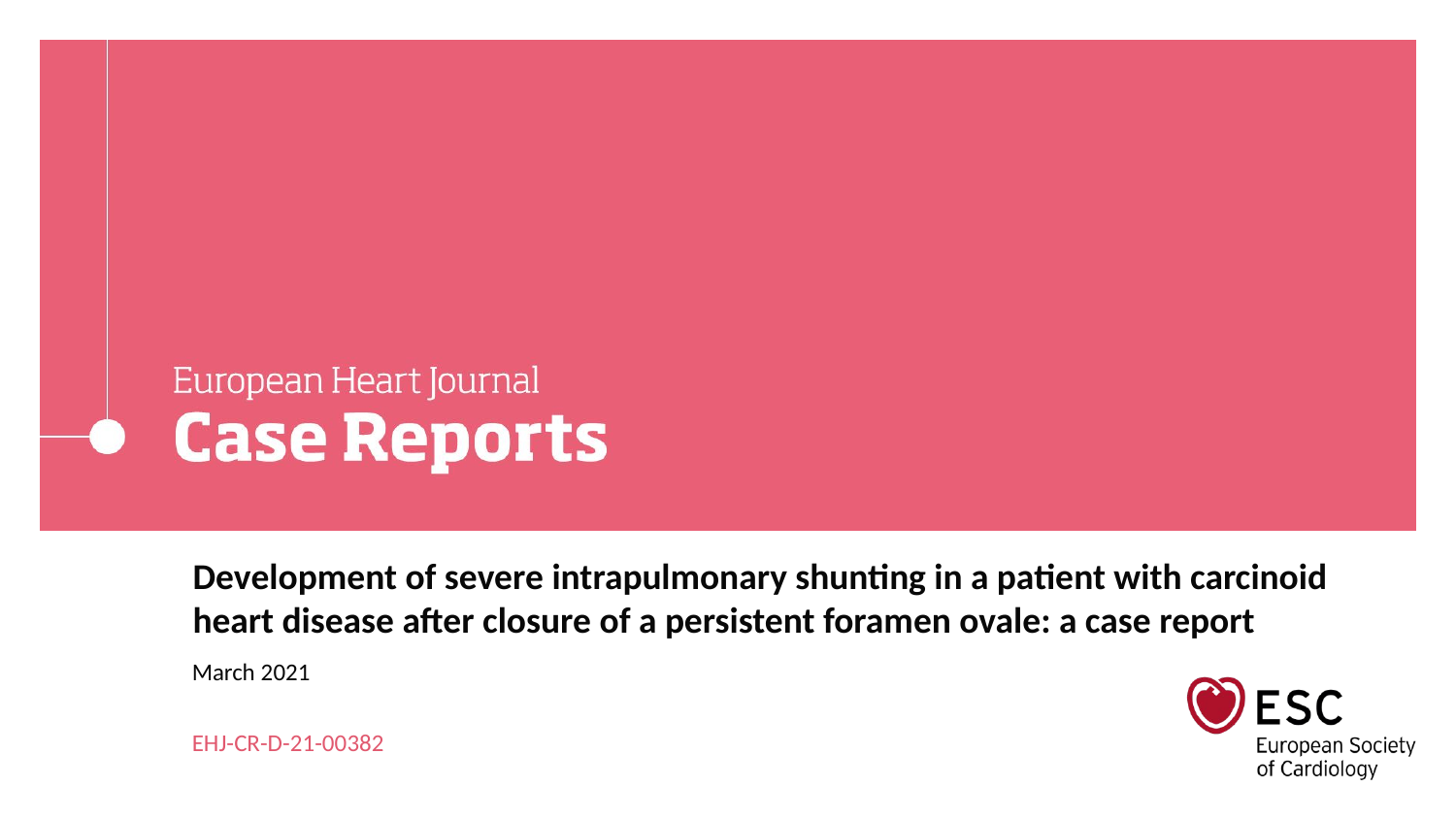

# Development of severe intrapulmonary shunting in a patient with carcinoid heart disease after closure of a persistent foramen ovale: a case report
March 2021
EHJ-CR-D-21-00382

## Slide 2
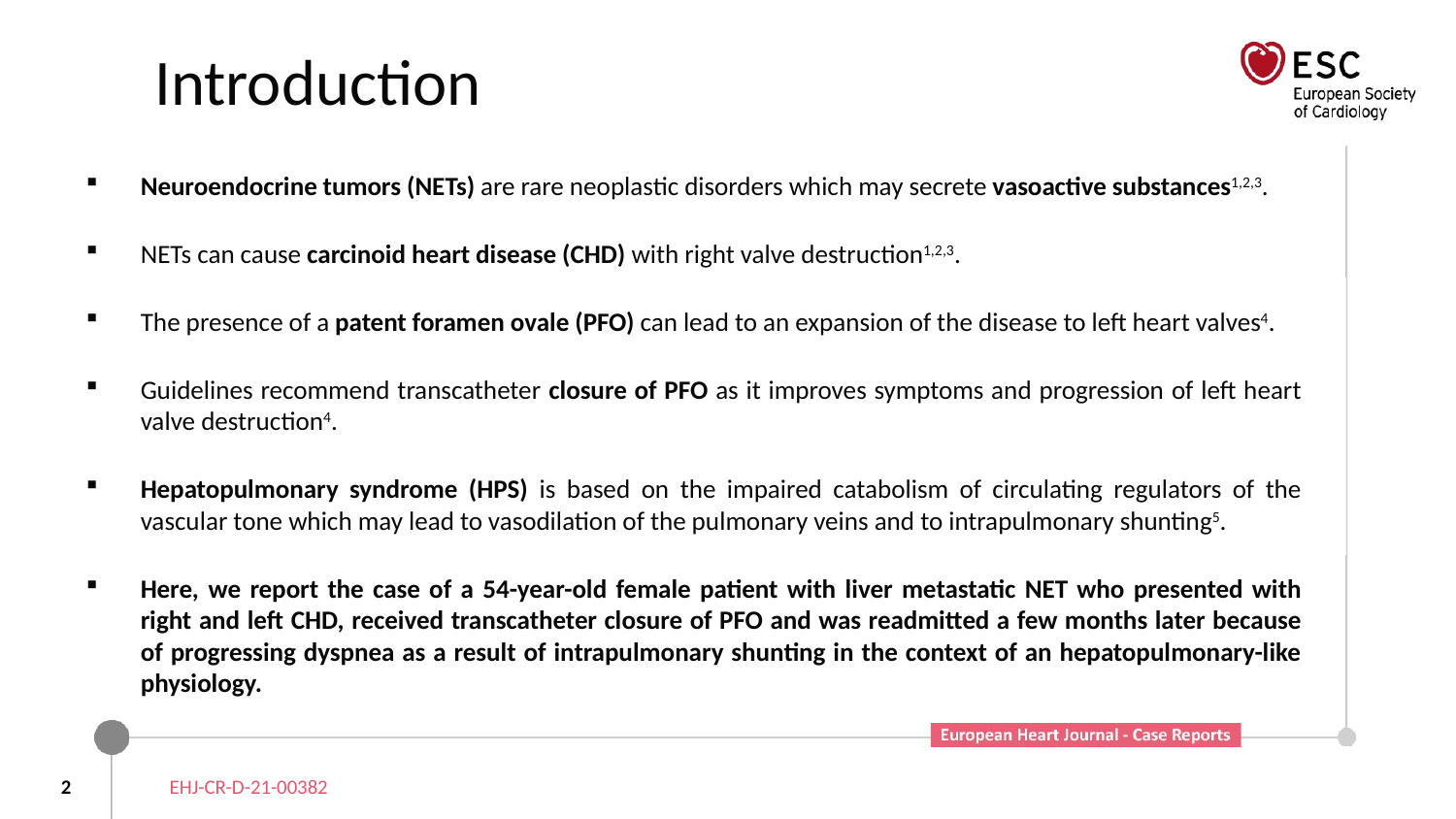

# Introduction
Neuroendocrine tumors (NETs) are rare neoplastic disorders which may secrete vasoactive substances1,2,3.
NETs can cause carcinoid heart disease (CHD) with right valve destruction1,2,3.
The presence of a patent foramen ovale (PFO) can lead to an expansion of the disease to left heart valves4.
Guidelines recommend transcatheter closure of PFO as it improves symptoms and progression of left heart valve destruction4.
Hepatopulmonary syndrome (HPS) is based on the impaired catabolism of circulating regulators of the vascular tone which may lead to vasodilation of the pulmonary veins and to intrapulmonary shunting5.
Here, we report the case of a 54-year-old female patient with liver metastatic NET who presented with right and left CHD, received transcatheter closure of PFO and was readmitted a few months later because of progressing dyspnea as a result of intrapulmonary shunting in the context of an hepatopulmonary-like physiology.
2
EHJ-CR-D-21-00382

## Slide 3
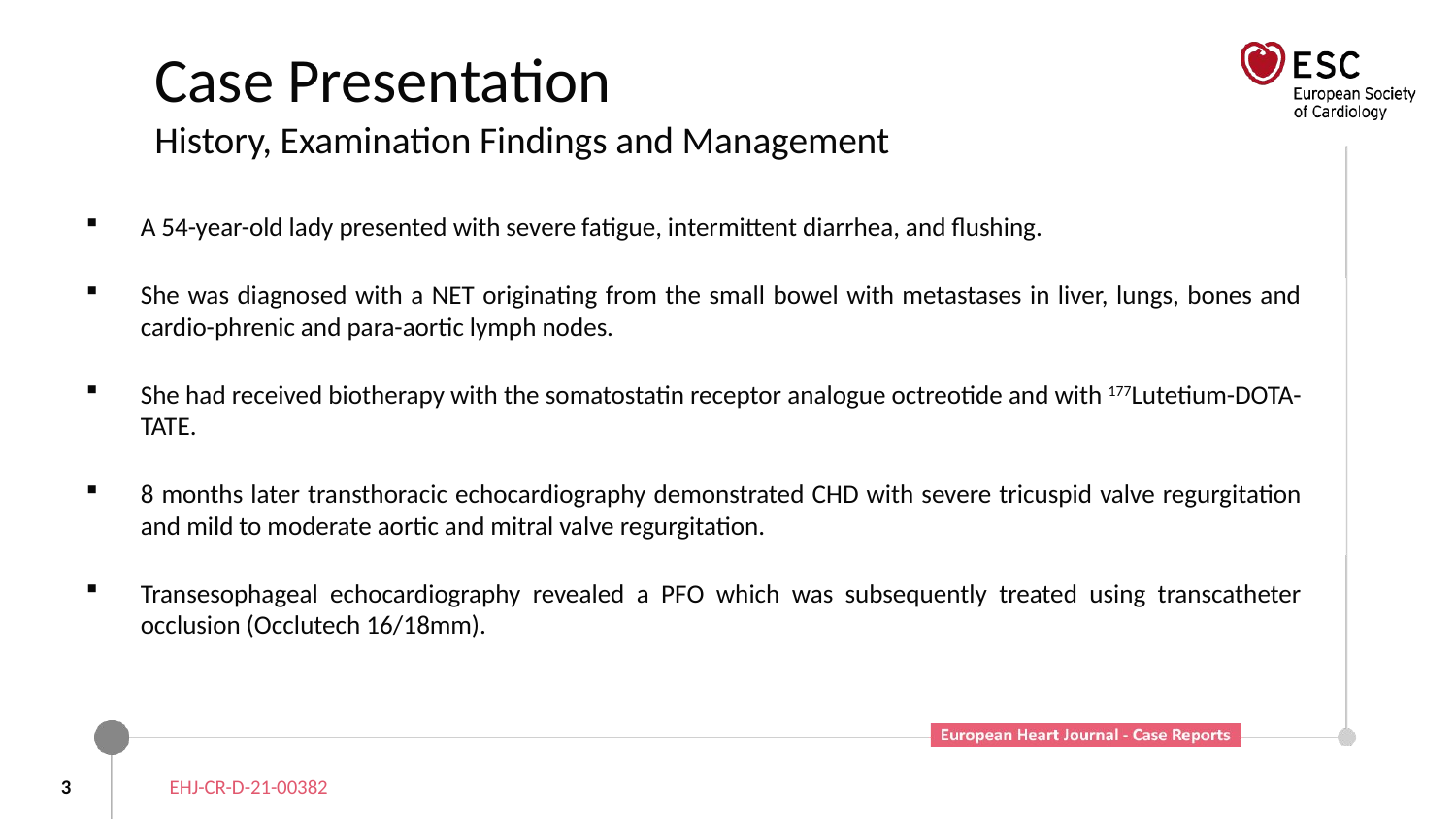

# Case Presentation History, Examination Findings and Management
A 54-year-old lady presented with severe fatigue, intermittent diarrhea, and flushing.
She was diagnosed with a NET originating from the small bowel with metastases in liver, lungs, bones and cardio-phrenic and para-aortic lymph nodes.
She had received biotherapy with the somatostatin receptor analogue octreotide and with 177Lutetium-DOTA-TATE.
8 months later transthoracic echocardiography demonstrated CHD with severe tricuspid valve regurgitation and mild to moderate aortic and mitral valve regurgitation.
Transesophageal echocardiography revealed a PFO which was subsequently treated using transcatheter occlusion (Occlutech 16/18mm).
3
EHJ-CR-D-21-00382

## Slide 4
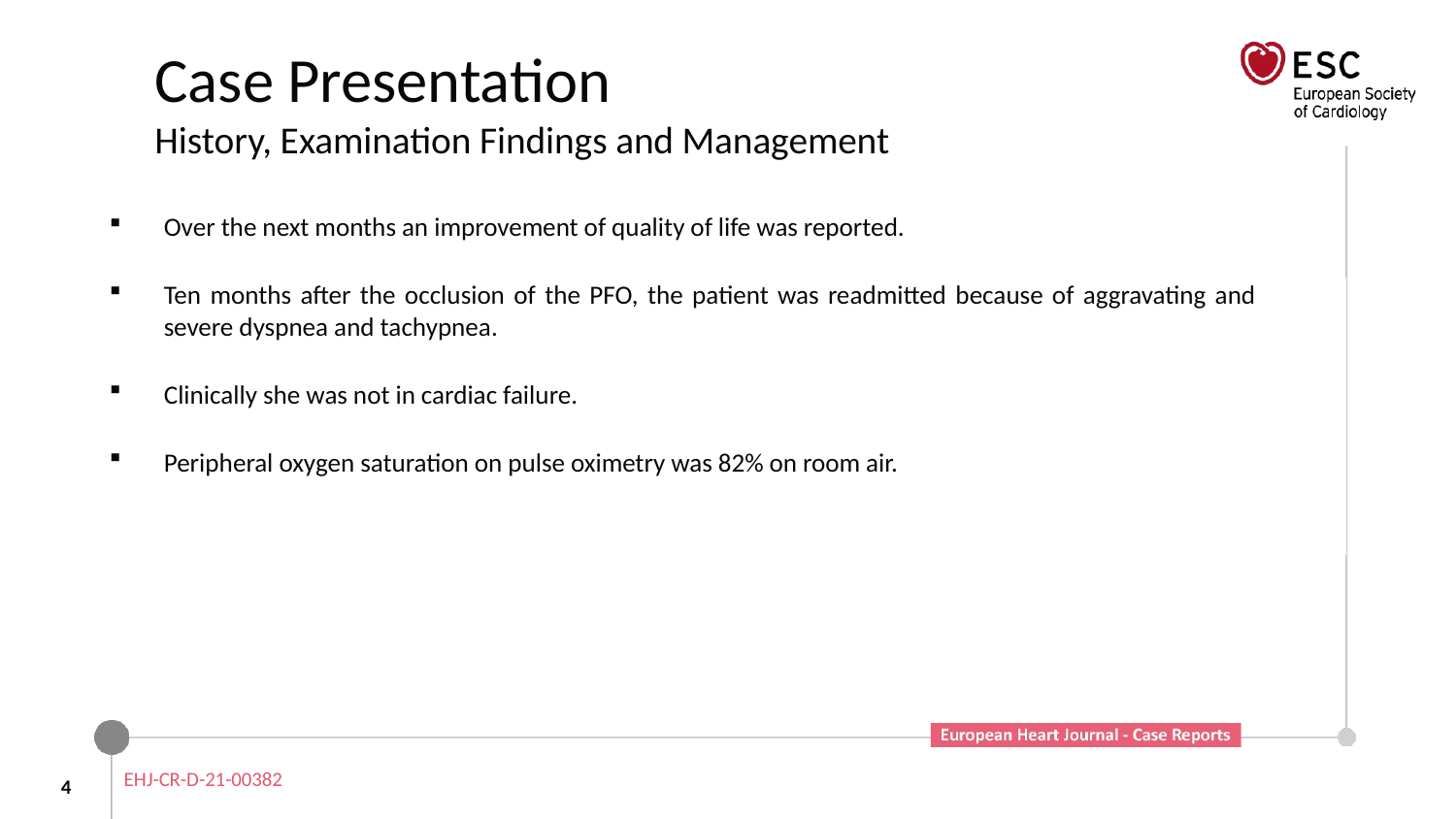

# Case Presentation History, Examination Findings and Management
Over the next months an improvement of quality of life was reported.
Ten months after the occlusion of the PFO, the patient was readmitted because of aggravating and severe dyspnea and tachypnea.
Clinically she was not in cardiac failure.
Peripheral oxygen saturation on pulse oximetry was 82% on room air.
EHJ-CR-D-21-00382
4

## Slide 5
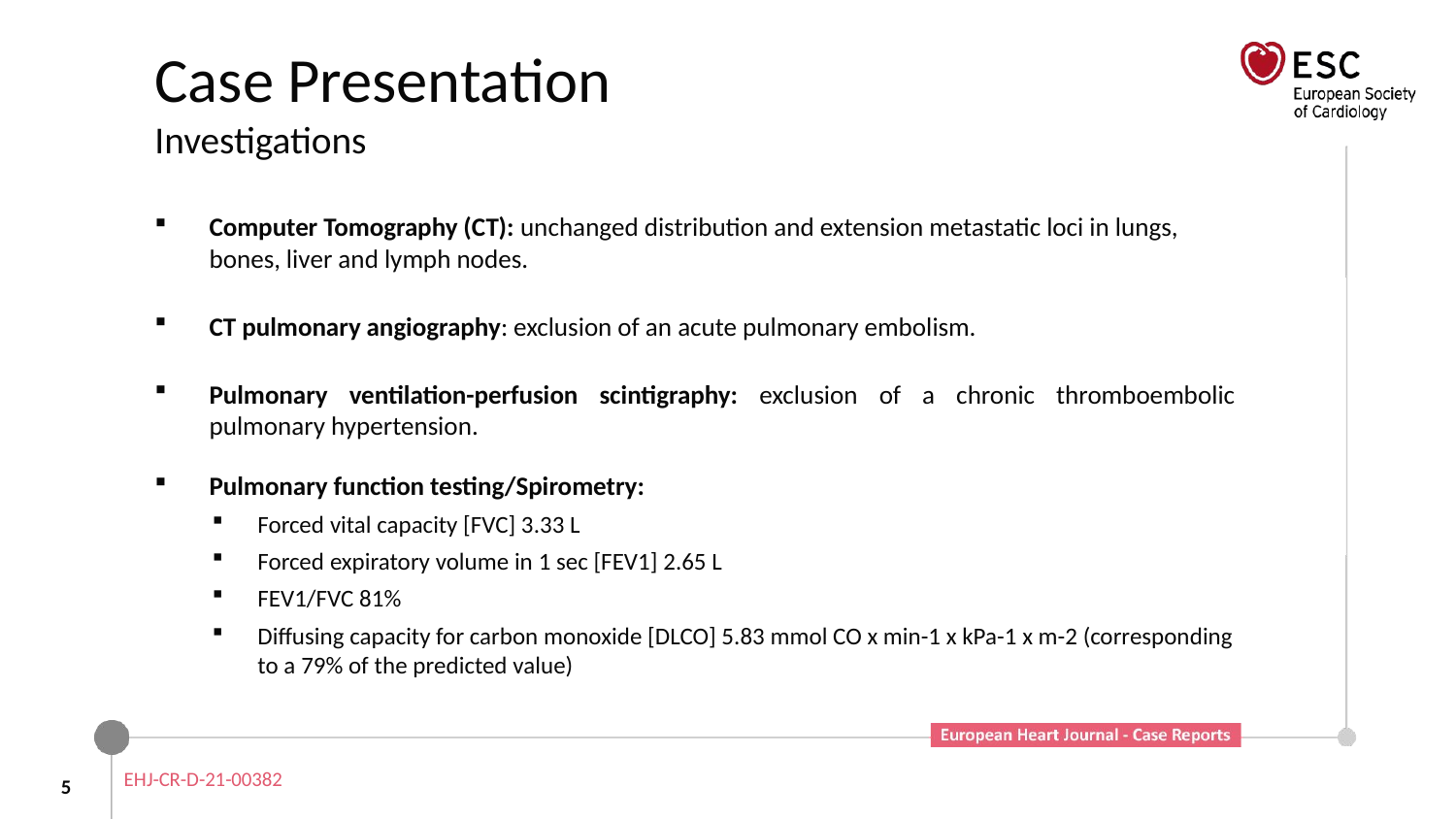

# Case Presentation Investigations
Computer Tomography (CT): unchanged distribution and extension metastatic loci in lungs, bones, liver and lymph nodes.
CT pulmonary angiography: exclusion of an acute pulmonary embolism.
Pulmonary ventilation-perfusion scintigraphy: exclusion of a chronic thromboembolic pulmonary hypertension.
Pulmonary function testing/Spirometry:
Forced vital capacity [FVC] 3.33 L
Forced expiratory volume in 1 sec [FEV1] 2.65 L
FEV1/FVC 81%
Diffusing capacity for carbon monoxide [DLCO] 5.83 mmol CO x min-1 x kPa-1 x m-2 (corresponding to a 79% of the predicted value)
EHJ-CR-D-21-00382
5

## Slide 6
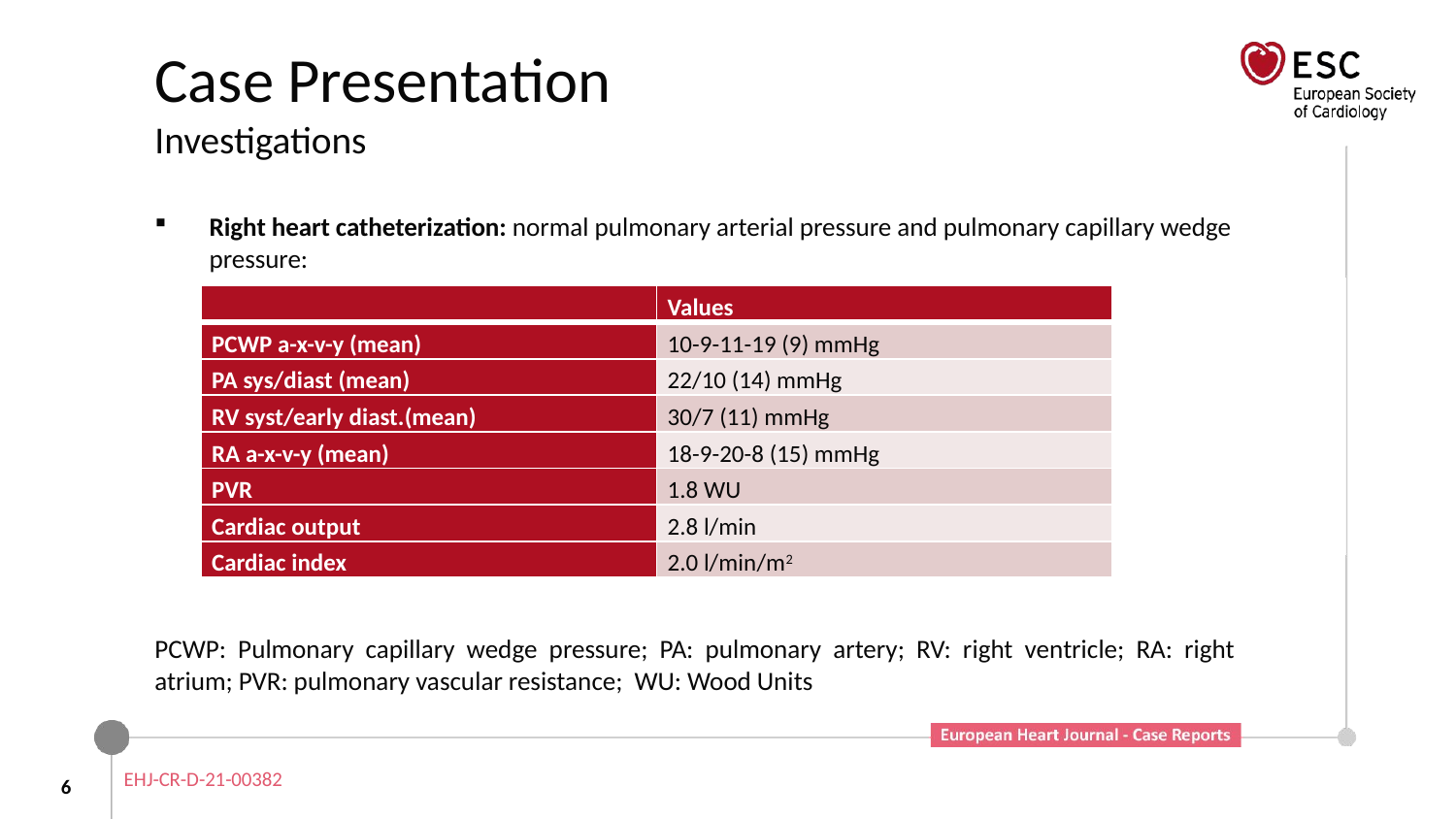

# Case Presentation Investigations
Right heart catheterization: normal pulmonary arterial pressure and pulmonary capillary wedge pressure:
PCWP: Pulmonary capillary wedge pressure; PA: pulmonary artery; RV: right ventricle; RA: right atrium; PVR: pulmonary vascular resistance; WU: Wood Units
| | Values |
| --- | --- |
| PCWP a-x-v-y (mean) | 10-9-11-19 (9) mmHg |
| PA sys/diast (mean) | 22/10 (14) mmHg |
| RV syst/early diast.(mean) | 30/7 (11) mmHg |
| RA a-x-v-y (mean) | 18-9-20-8 (15) mmHg |
| PVR | 1.8 WU |
| Cardiac output | 2.8 l/min |
| Cardiac index | 2.0 l/min/m2 |
EHJ-CR-D-21-00382
6

## Slide 7
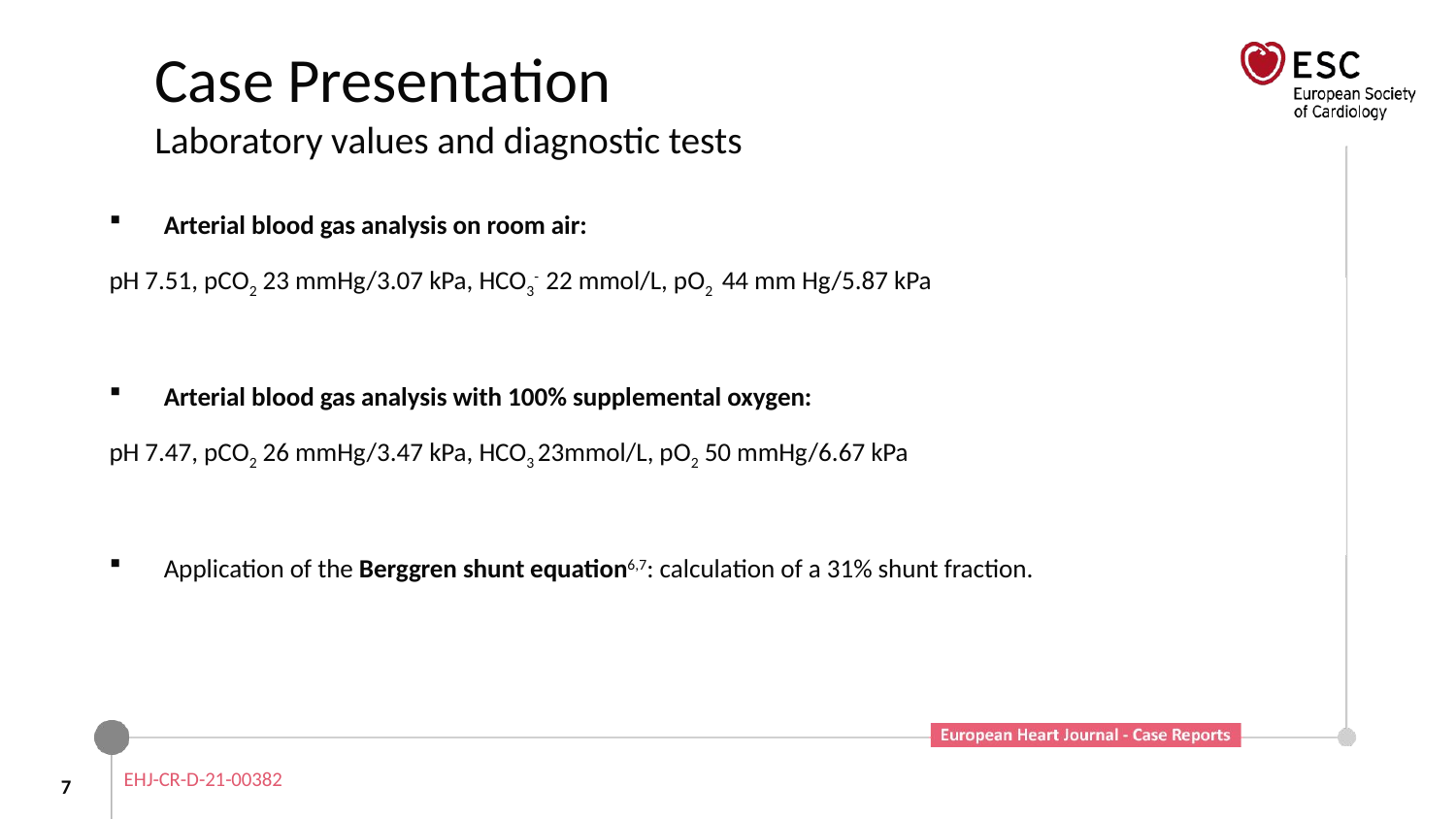

# Case Presentation Laboratory values and diagnostic tests
Arterial blood gas analysis on room air:
pH 7.51, pCO2 23 mmHg/3.07 kPa, HCO3- 22 mmol/L, pO2  44 mm Hg/5.87 kPa
Arterial blood gas analysis with 100% supplemental oxygen:
pH 7.47, pCO2 26 mmHg/3.47 kPa, HCO3 23mmol/L, pO2 50 mmHg/6.67 kPa
Application of the Berggren shunt equation6,7: calculation of a 31% shunt fraction.
EHJ-CR-D-21-00382
7

## Slide 8
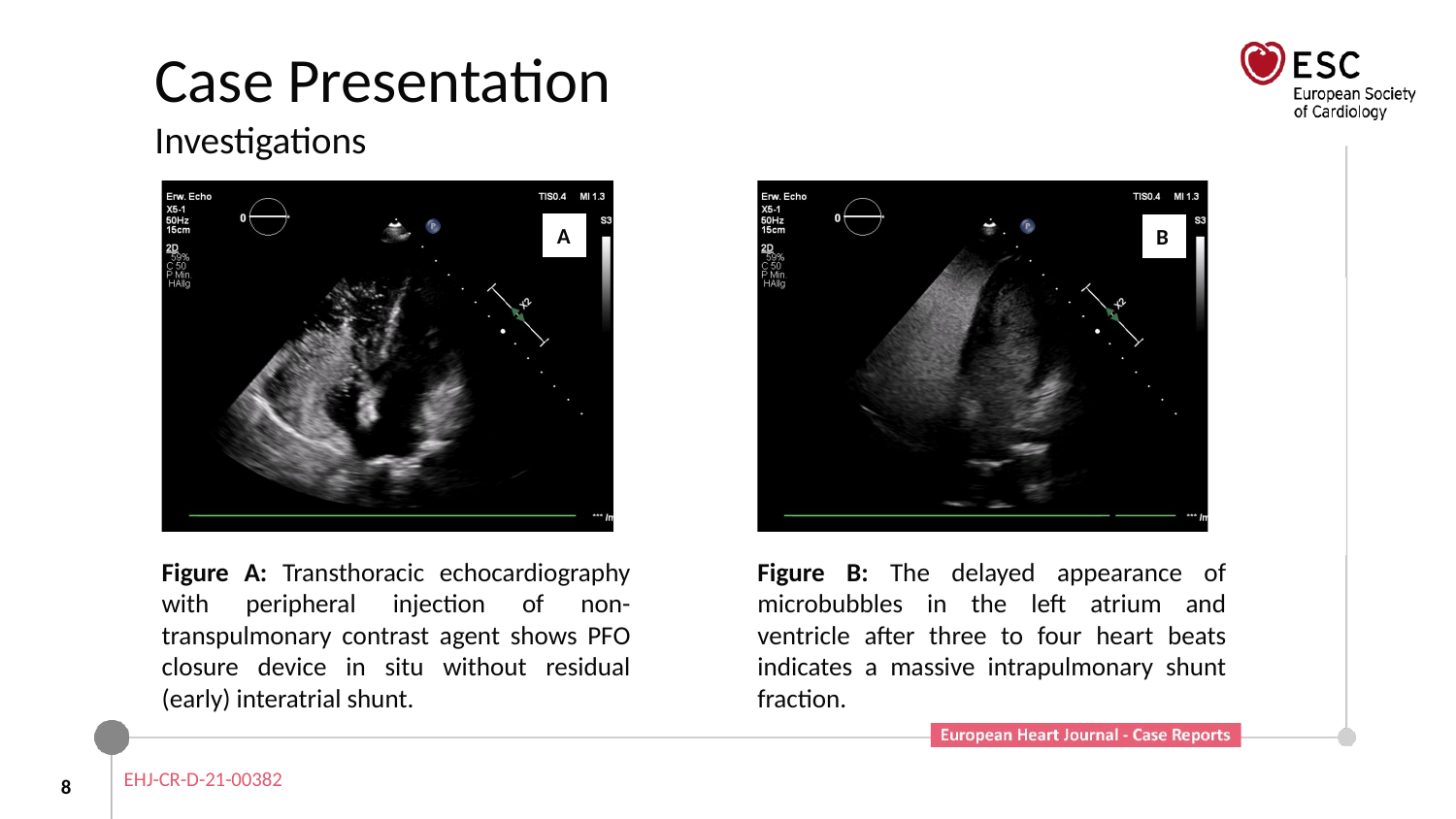

# Case Presentation Investigations
A
B
Figure A: Transthoracic echocardiography with peripheral injection of non-transpulmonary contrast agent shows PFO closure device in situ without residual (early) interatrial shunt.
Figure B: The delayed appearance of microbubbles in the left atrium and ventricle after three to four heart beats indicates a massive intrapulmonary shunt fraction.
EHJ-CR-D-21-00382
8

## Slide 9
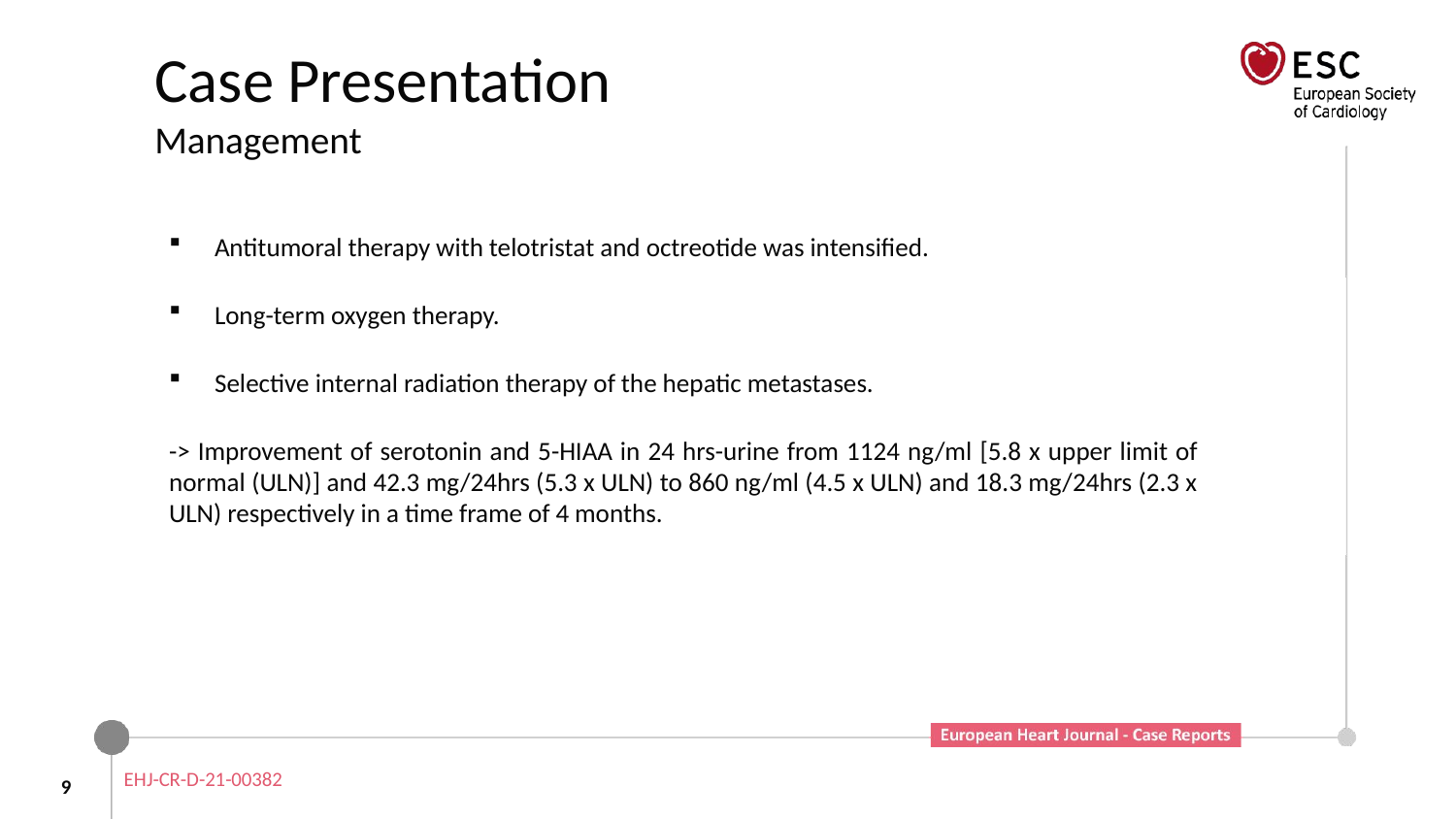

# Case Presentation Management
Antitumoral therapy with telotristat and octreotide was intensified.
Long-term oxygen therapy.
Selective internal radiation therapy of the hepatic metastases.
-> Improvement of serotonin and 5-HIAA in 24 hrs-urine from 1124 ng/ml [5.8 x upper limit of normal (ULN)] and 42.3 mg/24hrs (5.3 x ULN) to 860 ng/ml (4.5 x ULN) and 18.3 mg/24hrs (2.3 x ULN) respectively in a time frame of 4 months.
EHJ-CR-D-21-00382
9

## Slide 10
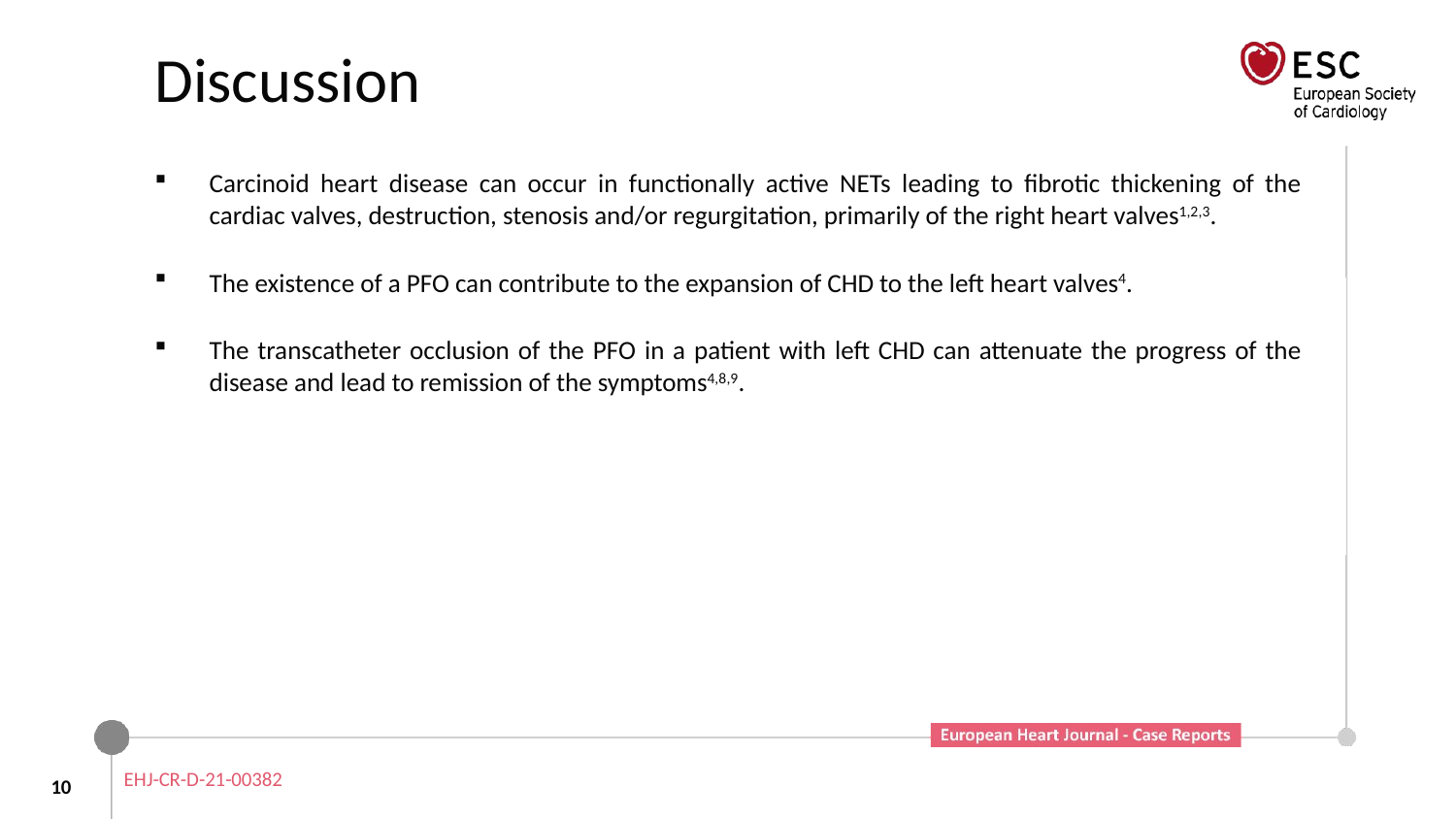

# Discussion
Carcinoid heart disease can occur in functionally active NETs leading to fibrotic thickening of the cardiac valves, destruction, stenosis and/or regurgitation, primarily of the right heart valves1,2,3.
The existence of a PFO can contribute to the expansion of CHD to the left heart valves4.
The transcatheter occlusion of the PFO in a patient with left CHD can attenuate the progress of the disease and lead to remission of the symptoms4,8,9.
EHJ-CR-D-21-00382
10

## Slide 11
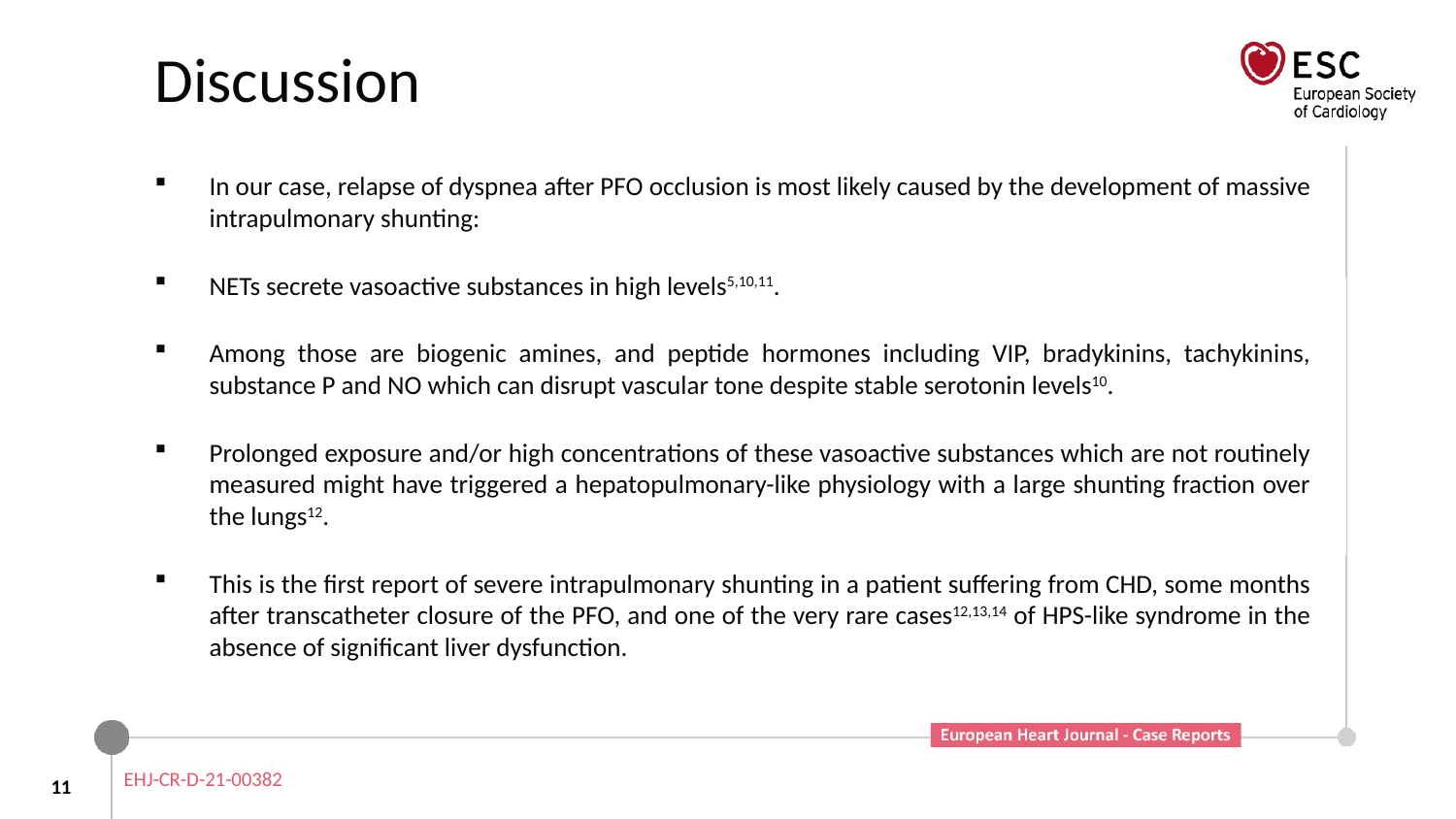

# Discussion
In our case, relapse of dyspnea after PFO occlusion is most likely caused by the development of massive intrapulmonary shunting:
NETs secrete vasoactive substances in high levels5,10,11.
Among those are biogenic amines, and peptide hormones including VIP, bradykinins, tachykinins, substance P and NO which can disrupt vascular tone despite stable serotonin levels10.
Prolonged exposure and/or high concentrations of these vasoactive substances which are not routinely measured might have triggered a hepatopulmonary-like physiology with a large shunting fraction over the lungs12.
This is the first report of severe intrapulmonary shunting in a patient suffering from CHD, some months after transcatheter closure of the PFO, and one of the very rare cases12,13,14 of HPS-like syndrome in the absence of significant liver dysfunction.
EHJ-CR-D-21-00382
11

## Slide 12
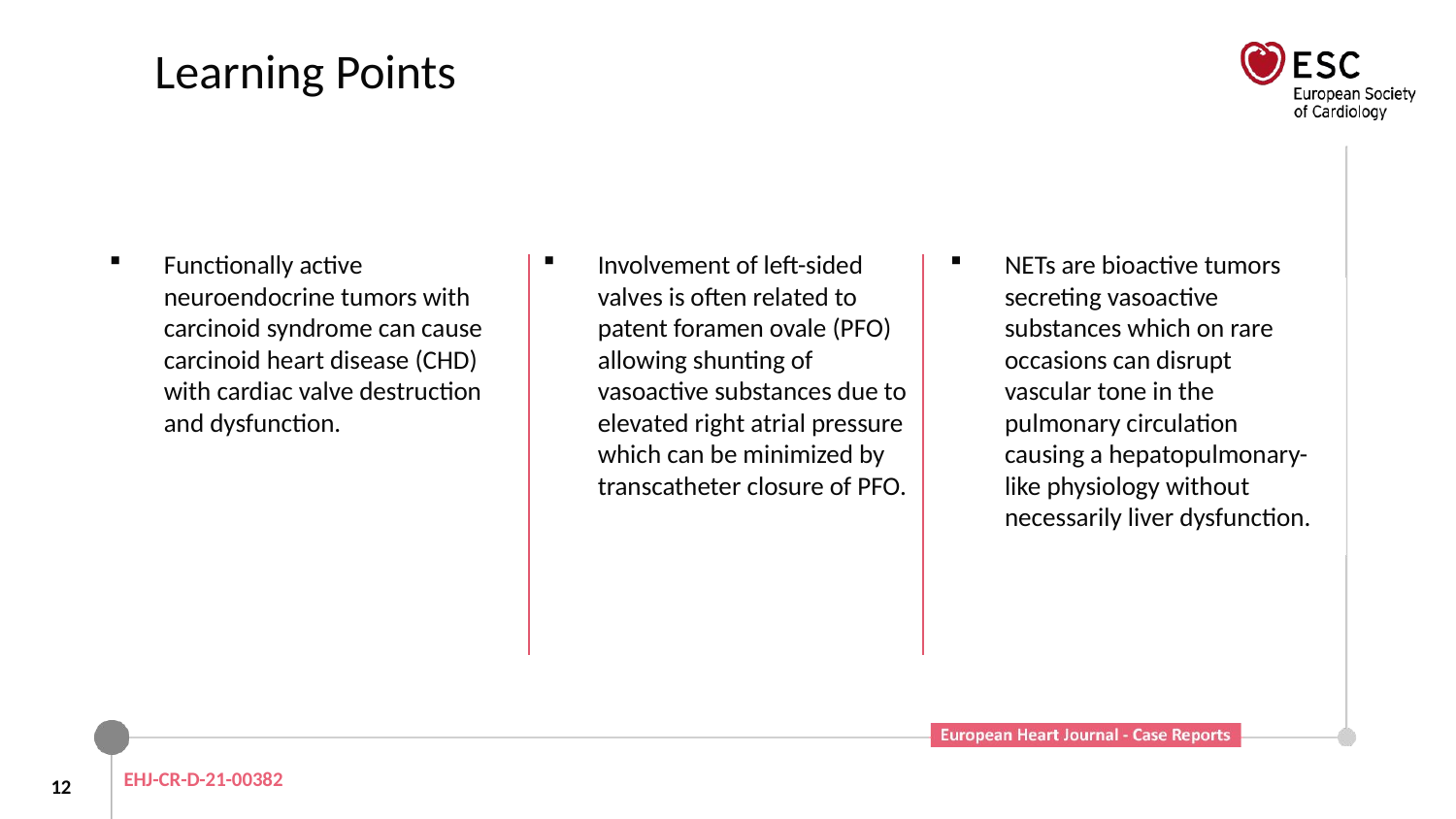

# Learning Points
NETs are bioactive tumors secreting vasoactive substances which on rare occasions can disrupt vascular tone in the pulmonary circulation causing a hepatopulmonary-like physiology without necessarily liver dysfunction.
Involvement of left-sided valves is often related to patent foramen ovale (PFO) allowing shunting of vasoactive substances due to elevated right atrial pressure which can be minimized by transcatheter closure of PFO.
Functionally active neuroendocrine tumors with carcinoid syndrome can cause carcinoid heart disease (CHD) with cardiac valve destruction and dysfunction.
EHJ-CR-D-21-00382
12

## Slide 13
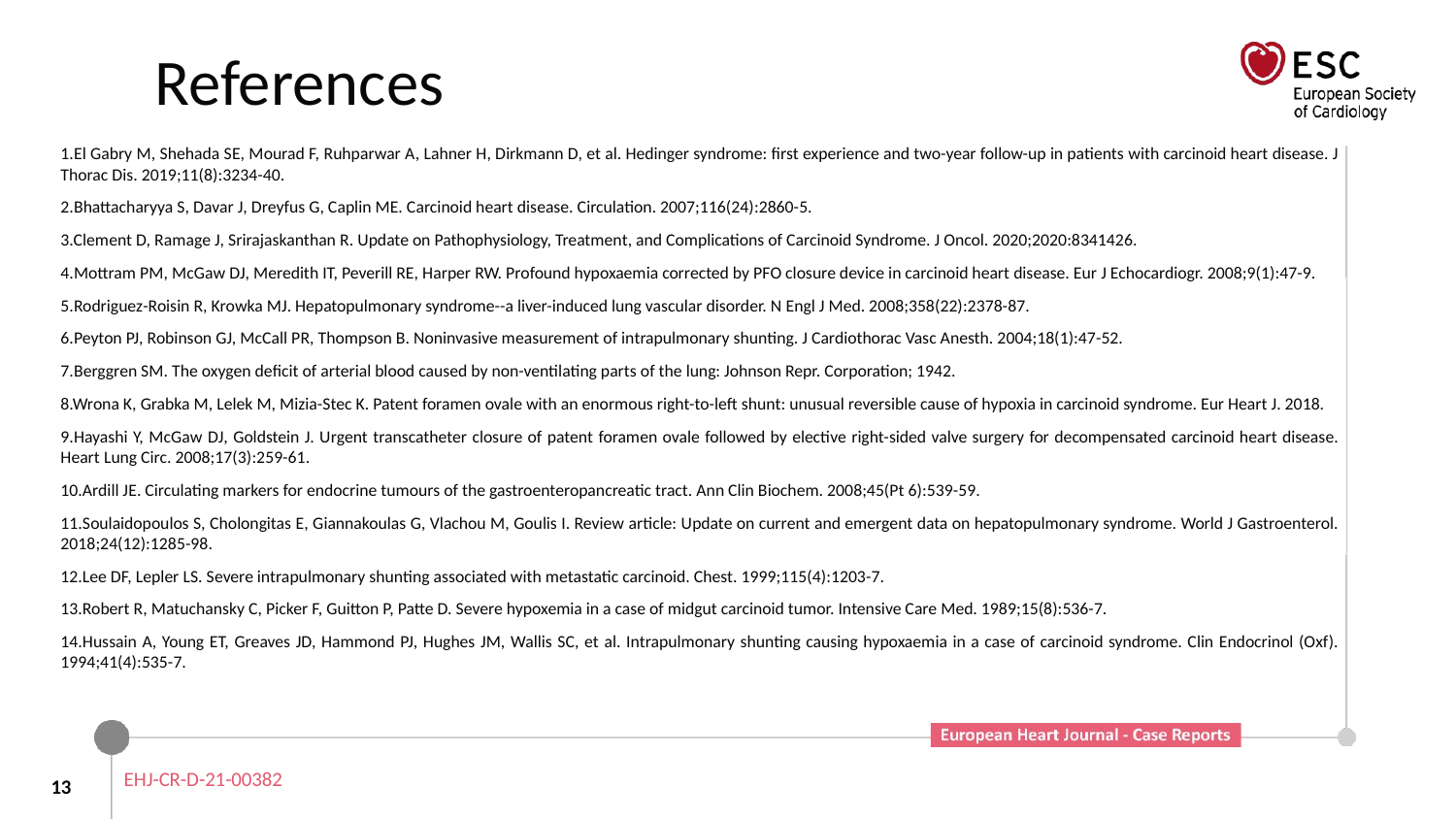

# References
1.El Gabry M, Shehada SE, Mourad F, Ruhparwar A, Lahner H, Dirkmann D, et al. Hedinger syndrome: first experience and two-year follow-up in patients with carcinoid heart disease. J Thorac Dis. 2019;11(8):3234-40.
2.Bhattacharyya S, Davar J, Dreyfus G, Caplin ME. Carcinoid heart disease. Circulation. 2007;116(24):2860-5.
3.Clement D, Ramage J, Srirajaskanthan R. Update on Pathophysiology, Treatment, and Complications of Carcinoid Syndrome. J Oncol. 2020;2020:8341426.
4.Mottram PM, McGaw DJ, Meredith IT, Peverill RE, Harper RW. Profound hypoxaemia corrected by PFO closure device in carcinoid heart disease. Eur J Echocardiogr. 2008;9(1):47-9.
5.Rodriguez-Roisin R, Krowka MJ. Hepatopulmonary syndrome--a liver-induced lung vascular disorder. N Engl J Med. 2008;358(22):2378-87.
6.Peyton PJ, Robinson GJ, McCall PR, Thompson B. Noninvasive measurement of intrapulmonary shunting. J Cardiothorac Vasc Anesth. 2004;18(1):47-52.
7.Berggren SM. The oxygen deficit of arterial blood caused by non-ventilating parts of the lung: Johnson Repr. Corporation; 1942.
8.Wrona K, Grabka M, Lelek M, Mizia-Stec K. Patent foramen ovale with an enormous right-to-left shunt: unusual reversible cause of hypoxia in carcinoid syndrome. Eur Heart J. 2018.
9.Hayashi Y, McGaw DJ, Goldstein J. Urgent transcatheter closure of patent foramen ovale followed by elective right-sided valve surgery for decompensated carcinoid heart disease. Heart Lung Circ. 2008;17(3):259-61.
10.Ardill JE. Circulating markers for endocrine tumours of the gastroenteropancreatic tract. Ann Clin Biochem. 2008;45(Pt 6):539-59.
11.Soulaidopoulos S, Cholongitas E, Giannakoulas G, Vlachou M, Goulis I. Review article: Update on current and emergent data on hepatopulmonary syndrome. World J Gastroenterol. 2018;24(12):1285-98.
12.Lee DF, Lepler LS. Severe intrapulmonary shunting associated with metastatic carcinoid. Chest. 1999;115(4):1203-7.
13.Robert R, Matuchansky C, Picker F, Guitton P, Patte D. Severe hypoxemia in a case of midgut carcinoid tumor. Intensive Care Med. 1989;15(8):536-7.
14.Hussain A, Young ET, Greaves JD, Hammond PJ, Hughes JM, Wallis SC, et al. Intrapulmonary shunting causing hypoxaemia in a case of carcinoid syndrome. Clin Endocrinol (Oxf). 1994;41(4):535-7.
EHJ-CR-D-21-00382
13
